# Interferon-Inducible Transmembrane Protein 3 Genetic Variant *rs12252* and Influenza Susceptibility and Severity: A Meta-Analysis

**DOI:** 10.1371/journal.pone.0124985

**Published:** 2015-05-05

**Authors:** Xianxian Yang, Bin Tan, Xipeng Zhou, Jian Xue, Xian Zhang, Peng Wang, Chuang Shao, Yingli Li, Chaorui Li, Huiming Xia, Jingfu Qiu

**Affiliations:** 1 School of Public Health and Management, Chongqing Medical University, Chongqing, China; 2 Department of inspection, The University-Town Affiliated Hospital of Chongqing Medical University, Chongqing, China; 3 School of Medicine, Johns Hopkins University, Baltimore, Maryland, United States of America; Washington University School of Medicine, UNITED STATES

## Abstract

**Background:**

The pandemic influenza A (H1N1) pdm09 virus, avian influenza A (H5N1) virus, and influenza A (H7N9) virus induced severe morbidity and mortality throughout the world. Previous studies suggested a close association between the interferon-induced transmembrane protein-3 (*IFITM3*) genetic variant *rs12252* and influenza. Here, we explored the correlation between the *rs12252* and influenza susceptibility and severity using meta-analysis.

**Methods:**

Relevant studies published before May 22, 2014 were retrieved from PubMed, ISI web of knowledge, EBSCO, and Cochrane central register of controlled trials databases. Association between *rs12252* and influenza susceptibility and severity were determined using statistical analysis of odds ratios (ORs).

**Results:**

A total of four studies consisting of 445 cases and 4180 controls were included in our analysis. Generally, there is increased risk of influenza in subjects carrying *rs12252* in the recessive model (CC vs. CT+TT: OR = 2.35, 95% CI: 1.49-3.70, *P*<0.001), the dominant model (CC+CT vs. TT: OR=1.60, 95% CI: 1.18–2.22, *P*=0.003), the homozygote comparison (CC vs. TT: OR=4.11, 95% CI: 2.15–7.84, *P*<0.001), and the allele contrast (C vs. T: OR=1.67, 95% CI: 1.32–2.13, *P*<0.001). Stratification analysis of ethnicity and severity revealed a significant increase in influenza susceptibility by *IFITM3-SNP rs12252 *among both Asian and Caucasian population. *SNP rs12252* shows significant impact on severe infections (*P*<0.05), but not on mild influenza. Besides, our result also associated *rs12252* with influenza severity (severe vs. mild: OR=2.37, 95% CI: 1.32–4.25, *P*=0.004), (severe vs. control: OR=2.70, 95% CI: 1.85–3.94, *P*<0.001).

**Conclusion:**

Our meta-analysis suggests a significant association between a minor *IFITM3* allele (*SNP rs12252-C*) with severe influenza susceptibility, but not in mild influenza subjects, in both UK Caucasians and Han Chinese population. The *rs12252*-C allele causes a 23.7% higher chance of infection and also constitutes a risk factor for more severe influenza.

## Introduction

In the past decade, emerging and re-emerging epidemic diseases including H7N9 virus in China and the Middle-East-Respiratory-Syndrome (MERS)-coronavirus in the Middle East and Europe have strongly resembled the threat caused by the Severe Acute Respiratory Syndrome (SARS)-coronavirus, H1N1 virus, and H5N1 virus to human health. Despite the effective vaccination, influenza remains a major global health threat due to the high morbidity and mortality caused by seasonal epidemics as well as the wide variety of the virus.

Accumulating evidence from murine and human samples revealed that influenza is the result of a combination of both host and viral genetic components. The risk factors of influenza included the intrinsic pathogenicity of the virus, acquired host factors (such as immunity and comorbidity) and intrinsic host susceptibility. The viral genetic determinants and host immunity have been widely studied, while the host genetic determinants remain elusive [1]. The study of host genetic factors involved in susceptibility to influenza is a promising strategy that may identify potential therapeutic targets [2]. As a result, systematical investigation of the association between host genetic factors and the occurrence of influenza is of pivotal importance. In 2009, the World Health Organization (WHO) identified studies of the role of host genetic factors on susceptibility to severe influenza as a priority [3].

Interferon-induced transmembrane proteins (*IFITMs*) inhibit infection of diverse enveloped viruses, including the influenza A virus (IAV), which was thought to enter from late endosomes [4,5,6]. Interferon-induced transmembrane protein-1 (*IFITM1)*, interferon-induced transmembrane protein-2 (*IFITM2)*, and *IFITM3* comprise a family of restriction factors that possess broad antiviral activities [6,7,8,9]. *IFITM3* is considered to be most active against IAV and resides in late endosomes and lysosomes using several orthologous genetic approaches [6,8,10,11]. Although there is a lack of direct evidence for full understanding of the molecular mechanism underlying *IFITM* protein and virus infection interaction, Everitt et al. has shown that severity of influenza virus infection was greatly increased in *IFITM3* knockout mice compared with wild-type animals. In humans, the rare *SNP rs12252-C* allele of *IFITM3* results in a truncated protein, leading to reduced restriction of virus replication in vitro. The *IFITM3 SNP rs12252-C* was strongly associated with worse clinical outcomes for patients infected with the 2009 pandemic IAV [12]. Wang et al. found that patients with the *rs12252-C/C IFN-IFITM3* genotype had more rapid disease progression and were less likely to survive [13].

So far, there were many studies which have provided evidence for the association between the *rs12252-C/C* genotype of *IFITM3* and severe influenza. However, these studies were seriously limited by their innate shortcomings. First, the majority of those studies were carried out on animal models instead of based on human clinical data and thus lack a translational stage in between. Secondly, these studies were seldom concerned with the association between *SNP rs12252* and susceptibility to mild or severe influenza. Finally, the sample sizes of these studies were relatively small, and the findings were inconsistent [12,13,14,15].

To resolve the controversy regarding the involvement of *IFITM3 SNP rs12252* in influenza, we conducted a systematic meta-analysis with characteristics of large sample sizes and two ethnic populations. This study should be helpful in the prevention and treatment of severe influenza as well as in studying host-virus interaction during pathogenesis in the future.

## Materials and Methods

### Data source

A literature search was conducted to looking for eligible studies that explored the association between *IFITM3 SNP rs12252* and influenza risk using PubMed, ISI web of knowledge, EBSCO, and Cochrane central register of controlled trials on May 22, 2014. The searching terms were: “influenza” combined with “interferon-inducible transmembrane protein” or “*IFITM3*” or “*rs12252*”. Moreover, we supplemented our search by screening the reference lists of all the relevant studies, including original articles, reviews, and meta-analyses. References to all identified publications were entered into reference-managing software (EndNote, version X6).

### Inclusion criteria

The initial screening of title and abstract was carried out by two reviewers independently (Xianxian Yang, Bin Tan). A second screening was based on full-text review by the same reviewers. Then the finally included studies whether they were in accordance with the method of cross-check were compared. Disagreements were discussed, and the consensus was reached with a third party (Jingfu Qiu) being involved when necessary. A study was considered appropriate for the meta-analysis when it met the following criteria:

1. It was cohort study or case control study in design.
2. It evaluated the association of *IFITM3 SNP rs12252* and influenza risk.
3. Genotype frequencies in cases and controls were both available for calculating an odds ratio (OR) and corresponding 95% confidence interval (CI).
4. All patients were definitively diagnosed with influenza confirmed by viral genome polymerase chain reaction (PCR) assay, and able to give informed consent.
5. Published in the English language.

### Exclusion criteria

Studies met one or more of the following exclusion criteria were rejected:

1. Repeatedly published literature.
2. Studies based on animals or family or sibling pairs rather than general population.
3. Data came from reviews and abstracts.
4. Studies with insufficient data.

### Data extraction

Three reviewers (Xianxian Yang, Bin Tan and Xipeng Zhou) independently extracted relevant data according to the previously data extraction form. The extraction results were evaluated by other reviewers (Jingfu Qiu, Xian Zhang). Disagreements were resolved by discussion. The extracted data included: the first author, year of publication, country of origin, ethnicity of the study population, type of virus, genotyping methods, sample size, source of control, the genotype and allele frequencies of the *rs12252*, and information for Hardy—Weinberg equilibrium (HWE) in control group.

### Quality assessment

Quality assessment was conducted for each article according to a pre-designed evaluation form based on Critical Appraisal Skills Programme (CASP) for case—control study [16] and Strengthening the Reporting of Genetic Association studies (STREGA) [17] containing eleven items associated with valid data reported in the study. For each item, there are three degrees, “yes”(scored 2),“can't tell”(scored 1) or “no”(scored 0), after evaluating each item, a total score from 0 to 22 was reported for each article. Studies would be divided into 3 grades: Grade A (scored 15–22, high quality), Grade B (scored 8–14, medium quality), or Grade C (scored 0–7, inferior quality). Only the studies of Grade A or B would be included in the final analysis.

### Statistical analysis

The pooled OR with its 95%CI was calculated to evaluate the strength of association between the *IFITM3-SNP rs12252* and influenza susceptibility or severity in five different genetic models, and the Z test was used to determine the significance of the pooled OR. Cochran’s χ^*2*^ -based Q statistic test was performed to assess possible heterogeneity between the individual studies [18]. The fixed-effects model was applied to calculate the pooled OR with its 95% CI when there was no obvious heterogeneity between studies, otherwise, the random-effects model was used [19,20]. Sensitivity analyses were conducted by omitting certain studies each time, such as studies deviated from HWE or studies carry out in certain population. Moreover, we performed subgroup analysis stratified by ethnicity or severity for the disease. Publication bias analysis was performed by the funnel plot and Egger’s linear regression test [21]. Statistical analyses were done with Review Manager 5.1 and Stata/SE. All *P* values are two-sided, and *P*<0.05 was considered statistically significant.

## Results

### Literature search

**Fig 1:**
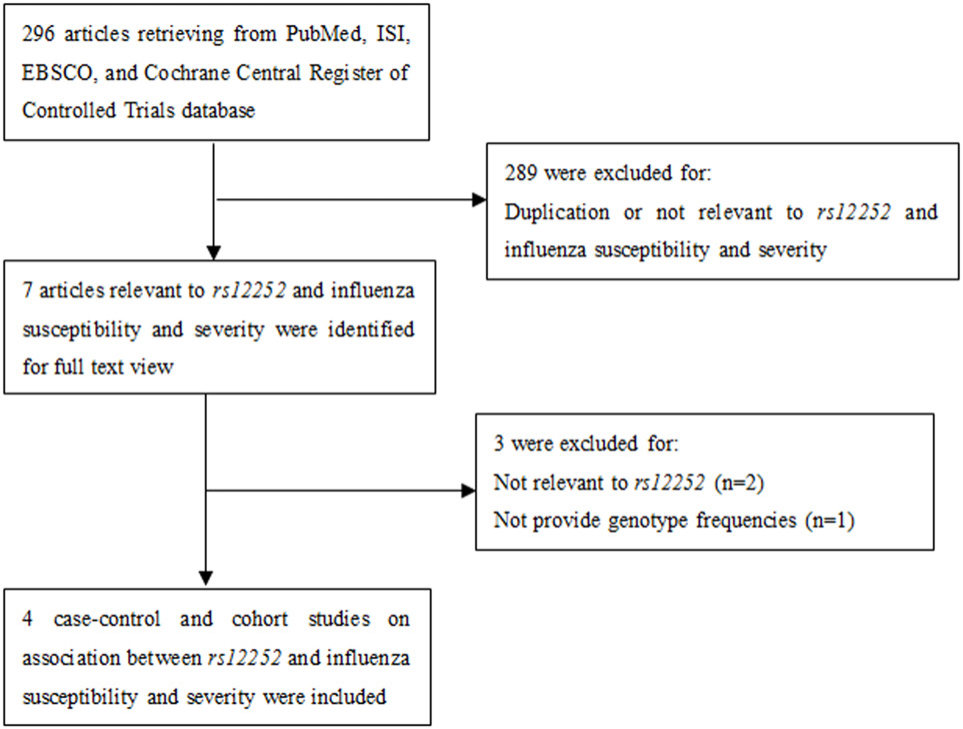
Flow chart of data selection.

A total of 296 potentially relevant publications up to May 22, 2014 were systematically identified through electronic databases. After screening the title and abstract, 7 studies were left excluding duplicates or those irrelevant to *SNP rs12252* of *IFITM3* and influenza risk or severity, reviews, and reports. Among them, 3 studies were excluded by full-text screening because they did not match the inclusion criteria described above. Finally, 4 studies [12,13,14,15] were included for the meta-analysis. The flow chart of article selection process is described in Fig 1.

### Studies characteristics

The basic characteristics of included studies are presented in Table 1, including country of origin, ethnicity, type of virus, sample size, type of control, genotyping method, and deviation from HWE. All studies were published in English and they were published between 2013 and 2014. Two studies were conducted in China, two in the UK. Case—control study designs were used in 1 study while the other 3 studies used cohort designs. There were 2 studies involving Caucasian participants and two studies involving Asian participants. The sample sizes ranged from 16 to 2623. Most cases were diagnosed based on clinical, consolidation and infiltrates on chest X-ray, and laboratory examination. Controls were mainly healthy populations (the allele frequencies and genotypes obtained from the 1,000 genomes project) matched with cases in gender, age, ethnicity and residential area. Table 2 presents the genotype frequencies of these studies.

**Table 1:** Characteristics of studies included in the meta-analysis.

Study	Year	Country	Ethnicity	Type of virus	Case/control	Design	Genotyping methods	Type of control	HWE	Quality grade
Everitt et al.	2013	UK	Caucasian	H1N1 and seasonal influenza virus	53/360	Cohort study	PCR	the 1,000 genomes project	0.37	A (scored 16)
Mills et al.	2013	UK	Caucasian	H1N1	293/2623	Case-control study	PCR	NHC	1	A (scored 17)
Wang et al.	2014	China	Asian	H7N9	16/1000	Cohort study	PCR	the 1,000 genome project	NA	A (scored 15)
Zhang et al.	2013	China	Asian	H1N1	83/197	Cohort study	PCR	the 1,000 genomes project	NA	A (scored 15)

H1N1, pandemic influenza A; H7N9, influenza A virus subtype; NHC, normal health controls; HWE, Hardy—Weinberg equilibrium; PCR, polymerase chain reaction; NA, not available.

**Table 2:** Genotype and allele distributions of *IFITM3-SNP rs12252* in influenza and controls.

Locus	Study	Case			Control			Case		Control	
		CC	CT	TT	CC	CT	TT	C	T	C	T
**Total**	Everitt et al.	3	4	46	1	24	335	10	96	26	694
	Mills et al.	2	25	266	4	202	2417	29	557	210	5036
	Wang et al.	6	7	3	26	37	37	19	13	89	111
	Zhang et al.	35	39	9	50	98	49	109	57	198	196
**Severe infection**	Everitt et al.	3	4	46	1	24	335	10	96	26	694
	Mills et al.	0	3	31	4	202	2417	3	65	210	5036
	Wang et al.	6	7	3	26	37	37	19	13	89	111
	Zhang et al.	22	8	2	50	98	49	52	12	198	196
**Mild infection**	Mills et al.	2	22	235	4	202	2417	26	492	210	5036
	Zhang et al.	13	31	7	50	98	49	57	45	198	196

### Quantitative synthesis

#### Meta-analysis of the *SNP rs12252* variant and influenza susceptibility

**Fig 2:**
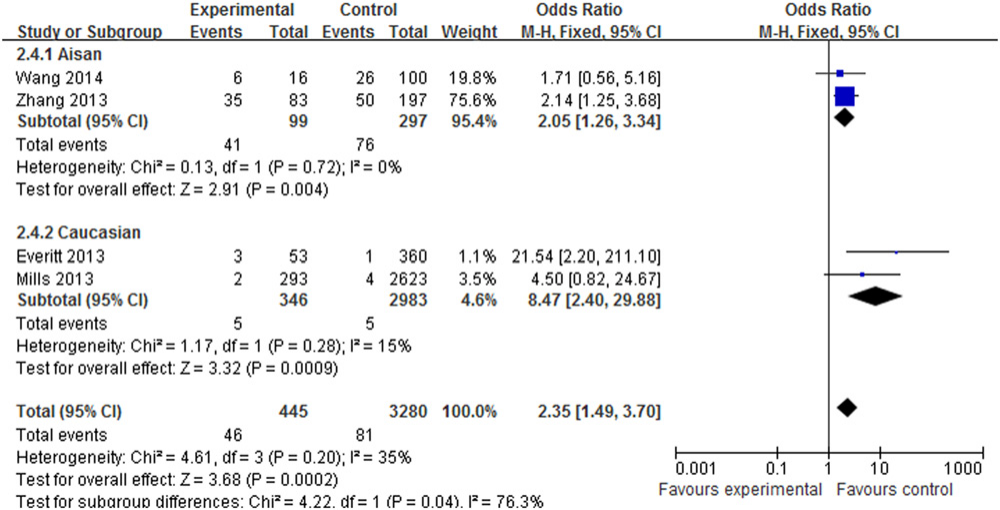
The forest plot for the association between *SNP rs12252* of *IFITM3* and influenza susceptibility in the recessive model (CC vs. CT+TT), subgroup analysis by different ethnicity.

**Fig 3:**
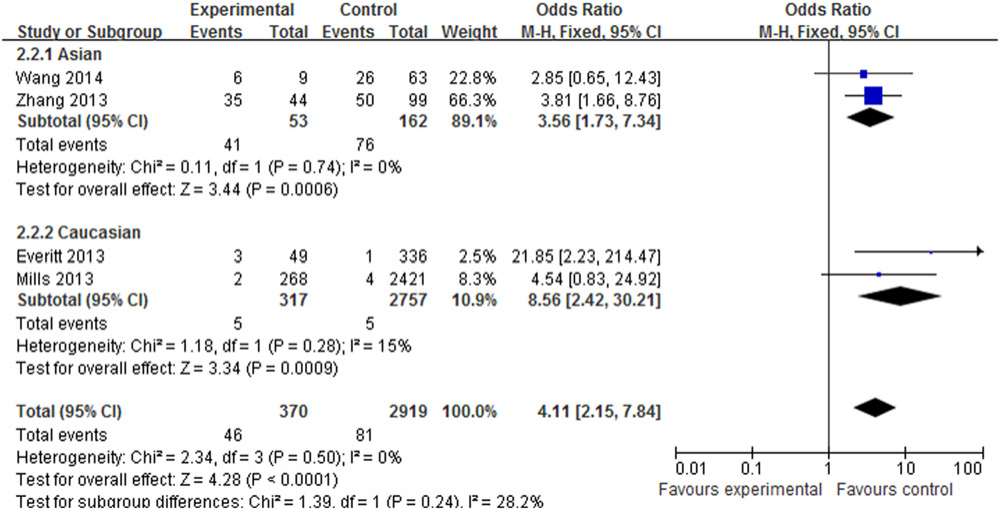
The forest plot for the association between *SNP rs12252* of *IFITM3* and influenza susceptibility in the homozygote comparison (CC vs. TT), subgroup analysis by different ethnicity.

**Fig 4:**
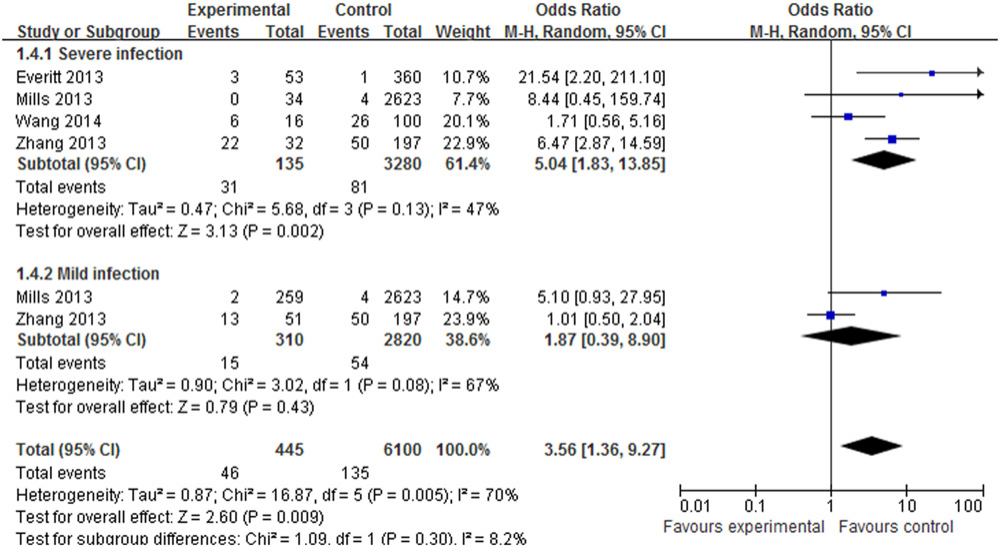
The forest plot for the association between *SNP rs12252* of *IFITM*3 and influenza susceptibility in the recessive model (CC vs. CT+TT), subgroup analysis by different severity. The individual block squares denote the susceptibility for each study published during 2014, with an area proportional to the amount of statistical information in each study. The horizontal line denotes a 95% confidence interval (CI). The pooled estimate and its 95% CI are represented by a diamond.

**Fig 5:**
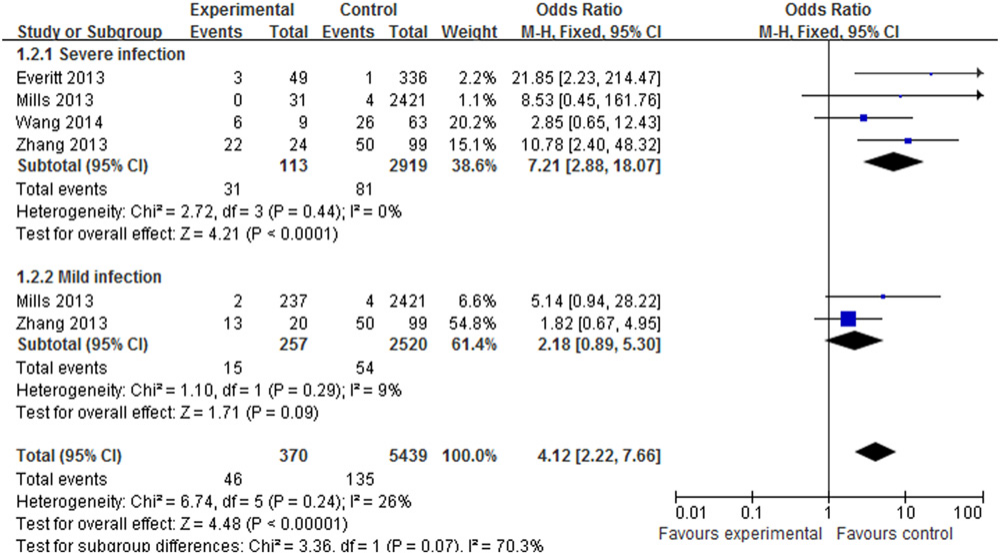
The forest plot for the association between *SNP rs12252* of *IFITM3* and influenza susceptibility in the homozygote comparison (CC vs. TT), subgroup analysis by different severity.

About 4 datasets involved in 445 cases and 4180 controls were included in the meta-analysis to explore the association of *rs12252* and influenza risk. There was no obvious heterogeneity between the individual studies using five genetic models (*P*>0.05). In overall analysis, a significant increase in influenza susceptibility was found in the recessive model (CC vs. CT+TT: OR = 2.35, 95% CI: 1.49–3.70, *P*<0.001), the dominant model (CC+CT vs. TT: OR = 1.60, 95% CI: 1.18–2.22, *P* = 0.003), the homozygote comparison (CC vs. TT: OR = 4.11, 95% CI: 2.15–7.84, *P*<0.001) and the allele contrast (C vs. T: OR = 1.67, 95% CI: 1.32–2.13, *P*<0.001), but not for the heterozygote comparison (CT vs. TT: OR = 1.37, 95% CI: 0.98–1.92, *P* = 0.07). In the subgroup analysis stratified by ethnicity, a significant increase in influenza susceptibility was found in Asian population subjects in the dominant model (CC+CT vs. TT: OR = 2.68, 95% CI: 1.38–5.18, *P* = 0.003), the recessive model (CC vs. CT+TT: OR = 2.05, 95% CI: 1.26–3.34, P = 0.004), the homozygote comparison (CC vs. TT: OR = 3.56, 95% CI: 1.73–7.34, *P*<0.001), the heterozygote comparison (CT vs. TT: OR = 2.20, 95% CI: 1.10–4.44, *P* = 0.03) and allele contrast (C vs. T: OR = 1.88, 95% CI: 1.34–2.63, *P*<0.001). A significant increase in influenza susceptibility was found in Caucasian population subjects in the recessive model (CC vs. CT+TT: OR = 8.47, 95% CI: 2.40–29.88, *P*<0.001) and the homozygote comparison (CC vs. TT: OR = 8.56, 95% CI: 2.42–30.21, *P*<0.001), but not for the dominant model, the heterozygote comparison and allele contrast. The details of the outcomes are shown in Table 3. The association between *rs12252* and influenza susceptibility stratified by ethnicity in the recessive model and the homozygote comparison respectively are shown in Figs 2 and 3. In the subgroup analysis stratified by severity, significantly increasing influenza susceptibility was found in severe infection subjects in the dominant model (CC + CT vs. TT: OR = 2.34, 95% CI: 1.35–4.04, *P* = 0.002), the recessive model (CC vs. CT+TT: OR = 5.04, 95% CI:1.83–13.85, *P*<0.001), the homozygote comparison (CC vs. TT: OR = 7.21, 95% CI: 2.88–18.07, *P*<0.001) and allele contrast (C vs. T: OR 2.70, 95% CI: 1.85–3.94, *P*<0.001), but not for the heterozygote comparison. No association was found between *rs12252* and mild influenza subjects. The details of these outcomes are shown in Table 3. The association between *rs12252* and influenza susceptibility stratified by severity in the recessive model and the homozygote comparison respectively are shown in Figs 4 and 5. Overall, our results suggest that patients with the *rs12252-C/C* genotype of *IFITM3* are more likely to have a significant increase in influenza susceptibility in both UK Caucasians and Han Chinese. In the subgroup analysis stratified by severity, the results suggest a significant association between a minor *IFITM3* allele (*SNP rs12252-C*) with severe influenza susceptibility, but not in mild influenza subjects.

**Table 3:** Meta-analysis of *IFITM3-SNP rs12252* and susceptibility of influenza.

Group	Genetic model	OR(95%CI)	*P*	Heterogeneity of study design			Effect modle
				Z	*P*_het_	*I* ^*2*^ *%*	
**Total**	CC+CT VS. TT	**1.61 [1.18, 2.22]**	0.003*	**2.97**	**0.21**	**34**	**F**
	CC VS. CT+TT	**2.35 [1.49, 3.70]**	< 0.001*	**3.68**	**0.20**	**35**	**F**
	CT VS. TT	1.37 [0.98, 1.92]	0.07	1.81	0.45	0	**F**
	CC VS. TT	**4.11 [2.15, 7.84]**	< 0.001*	**4.28**	**0.50**	**0**	**F**
	C VS. T	**1.67 [1.32, 2.13]**	< 0.001*	**4.21**	**0.24**	**30**	**F**
**Aisan**	CC+CT VS. TT	**2.68 [1.38, 5.18]**	0.003*	**2.92**	**0.093**	**0**	**F**
	CC VS. CT+TT	**2.05 [1.26, 3.34]**	0.004*	**2.91**	**0.72**	**0**	**F**
	CT VS. TT	**2.20 [1.10, 4.44]**	0.03*	**2.22**	**0.93**	**0**	**F**
	CC VS. TT	**3.56 [1.73, 7.34]**	< 0.001*	**3.44**	**0.74**	**0**	**F**
	C VS. T	**1.88 [1.34, 2.63]**	< 0.001*	**3.67**	**0.93**	**0**	**F**
**Caucasian**	CC+CT VS. TT	1.30 [0.89, 1.90]	0.17	1.36	0.29	12	F
	CC VS. CT+TT	**8.47 [2.40, 29.88]**	< 0.001*	**3.32**	**0.28**	**15**	**F**
	CT VS. TT	1.14 [0.76, 1.70]	0.54	0.62	0.90	0	F
	CC VS. TT	**8.56 [2.42, 30.21]**	< 0.001*	**3.34**	**0.28**	**15**	**F**
	C VS. T	1.74 [0.80, 3.78]	0.16	1.40	0.07	70	R
**Severe infection**	CC+CT VS. TT	**2.34 [1.35, 4.04]**	0.002*	**3.04**	**0.47**	**0**	**F**
	CC VS. CT+TT	**5.04 [1.83, 13.85]**	< 0.001*	**5.01**	**0.13**	**47**	**F**
	CT VS. TT	1.52 [0.82, 2.82]	0.19	1.32	0.84	0	F
	CC VS. TT	**7.21 [2.88, 18.07]**	< 0.001*	**4.21**	**0.44**	**0**	**F**
	C VS. T	**2.70 [1.85, 3.94]**	< 0.001*	**5.14**	**0.16**	**42**	**F**
**Mild infection**	CC+CT VS. TT	1.37 [0.94, 2.02]	0.10	1.63	0.26	21	F
	CC VS. CT+TT	1.87 [0.39, 8.90]	0.43	0.79	0.08	67	R
	CT VS. TT	1.32 [0.89, 1.96]	0.17	1.38	0.18	44	F
	CC VS. TT	2.18 [0.89, 5.30]	0.09	1.71	0.29	9	F
	C VS. T	1.26 [0.93, 1.71]	0.13	1.50	0.97	0	F

OR, odds ratio; CI, confidence interval; F, fixed-effect model; R, random-effect model; *P*_het_, *P* value of heterogeneity.

* *P*<0.05 stands for significance.

#### Meta-analysis of the *SNP rs12252* variant and influenza severity

Considering the two different types of influenza (Mild and Severe), we then assessed the association of *rs12252* and two different types of influenza risk using an allele genetic model. About 2 datasets consisting of 83 mild/moderate influenza patients and 55 severe influenza patients were included in the meta-analysis to explore the association of *rs12252* and influenza severity risk. There was no obvious heterogeneity between the individual studies using five genetic models (*P*>0.05). As shown in Table 4, we analyzed the C allele (relative to the T allele) and influenza severity risk for 2 studies. Overall, comparison of C and T alleles generated a 23.7% increased risk for influenza severity (severe vs. mild: OR = 2.37, 95% CI: 1.32–4.25, *P* = 0.004), (severe vs. control: OR = 2.70, 95% CI: 1.85–3.94, *P*<0.001) and (mild vs. control: OR = 1.26, 95% CI: 0.93–1.71, *P* = 0.13). Similar trends persisted for both CC and TT genotype associations. Overall, our data clearly suggest that the *rs12252*-*C* allele causes a 23.7% higher chance of infection and also constitutes a risk factor for more severe influenza.

**Table 4:** Meta-analysis of *IFITM3-SNP rs12252* and severity of influenza (C vs. T).

Comparison	OR(95%CI)	*P*	Heterogeneity of study design			Effect modle	Test of publication	
							bias	
			Z	*P*_het_	*I* ^*2*^ *%*		t value	*P* value
**Severe vs Mild**	**2.37 [1.32, 4.25]**	0.004*	**2.90**	**0.06**	**71**	R	0	1
Mild vs Control	1.26 [0.93, 1.71]	0.13	1.50	0.97	0	F	0	1
**Severe vs Control**	**2.70 [1.85, 3.94]**	< 0.000*	**5.14**	**0.16**	**42**	**F**	0.34	0.734

OR, odds ratio; CI, confidence interval; F, fixed-effect model; R, random-effect model; *P*_het_, *P* value of heterogeneity.

* *P*<0.05 stands for significance.

### Sensitivity analysis

Sensitivity analyses were conducted by omitting individual studies one by one sequentially and through the comparison between the results of pooled ORs for the random effects model and fixed effects model. We found that the results were not materially changed in other genetic models for *SNP rs12252* and influenza susceptibility, and sometimes the indicators for heterogeneity were reduced.

### Publication bias

**Fig 6:**
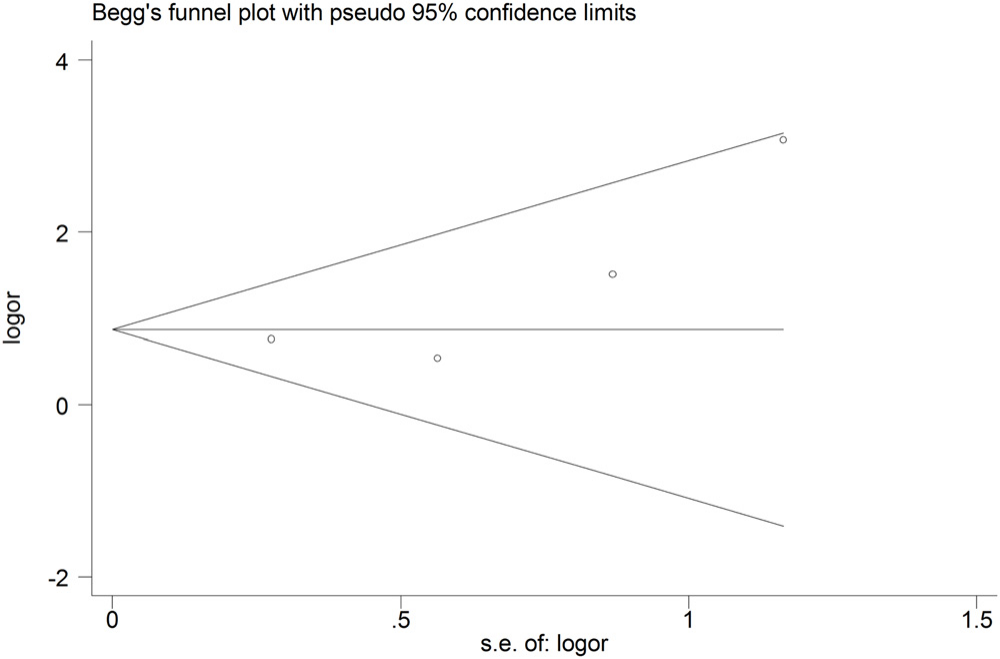
Begg’s funnel plot for publication bias in the recessive model (CC vs. CT+TT) for influenza.

A funnel plot of these four included studies was symmetrical and didn’t suggest a possibility of publication bias (Fig 6). The statistical results from Egger’s test still did not show publication bias for *IFITM3 SNP rs12252* (for C vs. T: *P*_egger_ = 0.348; for CC vs. TT: *P*_egger_ = 0.374; for CT vs. TT: *P*_egger_ = 0.314; for CC + CT vs. TT: *P*_egger_ = 0.151; for CC vs. CT + TT: *P*_egger_ = 0.241) in all five genetic models.

## Discussion

One of the main objectives of clinical research is to obtain clear and reliable results that can be used in patient management and serve as a basis for clinical guidelines. However, clinical trials do not always achieve this target and usually obtain controversial results. Meta-analysis, if properly applied, can contribute to this goal because of its strength in statistical comparison between studies and its ability to expand sample size and ethnic groups across studies. Thus, it has been widely adopted recently in various research fields worldwide.

There is no published meta-analysis so far for *rs12252* and influenza risk and severity studies. To explore the effect by *rs12252* on influenza risk and severity, we conducted extensive statistical analysis of association using meta-analysis. Generally speaking, the 4 studies included in this meta-analysis were of high quality (CASP score There is no published meta-analysis so far for *rs12252* and influenza risk and severity studies. To explore the effect by *rs12252* on influenza risk and severity, we conducted extensive statistical analysis of association using meta-analysis. Generally speaking, the 4 studies included in this meta-analysis were of high quality (CASP score ≥ 15). All of the studies clearly stated the total sample sizes, inclusion criteria of subjects, characteristics of participants and genotyping methods. Moreover, cases and controls were matched in age and gender from the 1000 Genomes Project or normal health people.

The 2009 H1N1 influenza pandemic showed the speed that a novel respiratory virus can spread and the ability of a generally mild infection to induce severe morbidity and mortality. In March 2013, an influenza outbreak caused by an unique avian origin H7N9 influenza A virus emerged in the Yangtze River Delta on China’s eastern seaboard [22,23,24,25,26]. The H7N9 virus has caused 134 confirmed cases in nine provinces, and mortality up to 30% [27,28]. The influenza pandemic is a serious health problem and is very difficult to cure. Recent in vitro studies show that the *IFITM* protein family members potently restrict the replication of multiple pathogenic viruses, including influenza viruses [7,10,29,30,31]. However, Everitt et al. showed that first, *IFITM3* knockout mice were found to develop fulminant viral pneumonia when challenged with an otherwise-low-pathogenicity H3N2 influenza A virus. Second, hospitalized patients who were severely ill from seasonal flu or 2009 H1N1 pandemic flu exhibited an enrichment of a minor *IFITM3* allele (*SNP rs12252-C*) that lacks the region corresponding to the first amino-terminal 21 amino acids due to the alteration of a splice acceptor site [12]. So we conducted this meta-analysis to confirm the association between the *IFITM3 SNP rs12252* and influenza susceptibility and severity. Although only four studies were included in this meta-analysis, it will provide the basis for further study of molecular epidemiology, while providing useful predictive measures of outcome that should be of help in managing severe influenza.

Although several studies [12,13,14,15] have investigated the association between *IFITM3 SNP rs12252* and influenza, the inconsistent results made the involvement of *IFITM3 SNP rs12252* in influenza inconclusive. Our meta-analysis included 445 patients with influenza and 4180 controls from 4 studies. We investigated the association between the *IFITM3 SNP rs12252* in general and also within different ethnic and severity subgroups. In addition, we studied *rs12252* and influenza severity in 83 mild/moderate and 55 severe influenza cases. Our data showed that patients with the *rs12252-C/C* genotype of *IFITM3* are more likely to increase the susceptibility to severe influenza but not in mild influenza, in both UK Caucasians and Han Chinese. We also found that the *rs12252*-C allele showed a 23.7% increased chance of infection along with a risk for severe influenza. Similar trends persisted for both CC and TT genotype associations. Considering the wider confidence intervals and small sample sizes of population-based studies, more studies are required to quantify the size of this effect reliably. Future studies will also ask whether the disease course differs between severely infected patients who have the CC genotype and those with the CT or TT genotypes. This largely depends on the frequency of the risk genotype in the population, so this should be explored in future epidemiological studies.

Recently, Williams et al. reported a putative association that *IFITM3* polymorphism *rs12252-C* restricts IAV [32]. However, our data suggest that patients with the *rs12252-C/C* genotype of *IFITM3* are more likely to attribute to the significant increase in severe influenza susceptibility in both UK Caucasians and Han Chinese. This may be explained as due to the different subjects since we use human data while Williams et al. utilized murine animals and cell culture.

It is intriguing that the CC genotype is rare in Northern Europeans [12] while common in Asian populations [15]. Our results imply that the incidence of severe influenza infection might be higher in Chinese than in Northern Europeans. Early screening for the *IFITM3* genotype might help to evaluate and predict the patient susceptibility and severity of disease thereby providing critical information for decision making during treatment.

We should also pay attention to the several limitations in our study, which may affect the result. First, we only included published English articles available from four databases. Relevant articles published in other languages, in other databases and unpublished studies may have been missed, which might bias the results. Secondly, only four studies (two for UK and two for Chinese) were included in this meta-analysis. Thus, data on Asian and Caucasian subjects are inadequate. Considering this fact, the results from these two ethnicities should be interpreted with caution. Therefore, additional research in these two ethnicities and in other ethnicities is needed to generalize the findings. Thirdly, some studies had small sample sizes, which may affect the statistical power of the publication bias. Although publication bias in the meta-analysis by Egger’s test was not significant, there is also relatively little bias in the summary effect size estimate, so the results of these tests must be interpreted with caution in small-sample meta-analyses. Finally, considering the complex genetic network within, the potential role of the *IFITM3 SNP rs12252* might be diluted or masked by other gene-gene or gene-environment interactions. Therefore, the jury must remain out before the eventual truth prevails.

## Conclusion

Overall, to the best of our knowledge, this is the first meta-analysis evaluating the association between *IFITM3 SNP rs12252* and influenza susceptibility and severity. Our meta-analysis suggests a significant association between a minor *IFITM3* allele (*SNP rs12252-C*) and severe influenza susceptibility, but not in mild influenza subjects, in both UK Caucasians and the Han Chinese population. The *rs12252*-C allele causes a 23.7% higher chance of infection and also constitutes a risk factor for more severe influenza. Our study exemplified a correlation study between *SNP rs12252* and influenza susceptibility and severity for future systemic epidemiological studies with larger sample sizes and more ethnicities included.
